# The Passing of Prejudice

**Published:** 1920-08-14

**Authors:** 


### The Passing of Prejudice.

Some most encouraging comments on the growing interest of parents in the health of their children are contained in the report of the Senior Medical Officer of Health for Nottingham (Dr. E. M. Wyche). He notes this as a gratifying circumstance in view of the earlier distrust with which the school medical service had to contend, and he says that to-day the medical officers' labours are greatly lightened by the parents' appreciation and the children's trust and confidence. Even in the dental side of the work Dr. Wyche is bold enough to speak of its "popularity," as evidenced by the steady increase of children seeking treatment, and, with regard to the recovery schools, Dr. Wyche says that if he were to select for admission all those children for whom their mothers make application, it would be necessary to double the accommodation. He is a warm advocate of the organised teaching of the principles and practice of hygiene in the schools, and of the great value of open-air education for combating the adverse effects of town-dwelling in early life he says it is difficult to speak too emphatically. The regular succession of rest and exercise, work and play, coupled with wholesome meals and the cultivation of the habit of cleanly open-air life, works wonders. In giving the statistics of the defects in Nottingham's school children, he says that much of it could, of course, be avoided by intelligent care and forethought. But he declares that we have yet to attain a wider knowledge of preventive medicine, and to see to it that the knowledge of hygiene, rightly taught, becomes the common knowledge of the people. We are breaking down prejudice. We must now build up knowledge.

